# Structure–Property Relationship in Isotactic Polypropylene Under Contrasting Processing Conditions

**DOI:** 10.3390/polym17141889

**Published:** 2025-07-08

**Authors:** Edin Suljovrujic, Dejan Milicevic, Katarina Djordjevic, Zorana Rogic Miladinovic, Georgi Stamboliev, Slobodanka Galovic

**Affiliations:** 1 Vinca Institute of Nuclear Sciences-National Institute of the Republic of Serbia, University of Belgrade, 11351 Belgrade, Serbia; dejanmilicevic@vinca.rs (D.M.); katarina.djordjevic@vin.bg.ac.rs (K.D.); zoranar@vinca.rs (Z.R.M.); gstambol@hotmail.com (G.S.); 2 Global Supply Line, Adelaide 5109, Australia

**Keywords:** semicrystalline polymers, polypropylene, processing conditions, crystallization, polymorphism

## Abstract

Polypropylene (PP), with its good physical, thermal, and mechanical properties and excellent processing capabilities, has become one of the most used synthetic polymers. It is known that the overall properties of semicrystalline polymers, including PP, are governed by morphology, which is influenced by the crystallization behavior of the polymer under specific conditions. The most important industrial PP remains the isotactic one, and it has been studied extensively for its polymorphic characteristics and crystallization behavior for over half a century. Due to its regular chain structure, isotactic polypropylene (iPP) belongs to the group of polymers with a high tendency for crystallization. The rapid quenching of molten iPP fails to produce a completely amorphous polymer but leads to an intermediate crystalline order. On the other hand, slow cooling yields a material with high crystalline content. The processing conditions that occur in practice and industry are between these two extremes and, in some cases, are even very close. Therefore, the study of limits in processability and the impact of extreme preparation conditions on morphology, structure, thermal, and mechanical properties fills a gap in the current understanding of how the processing conditions of iPP can be used to design the desired properties for specific applications and is in the focus of this research. The first set of samples (Q samples) was obtained by rapid quenching, while the second was prepared by very slow cooling from the melt to room temperature (SC samples). Testing of samples was performed by optical microscopy (OM), scanning electron microscopy (SEM), wide-angle X-ray diffraction (WAXD), Fourier transform infrared spectroscopy (FTIR), differential scanning calorimetry (DSC), dynamic dielectric spectroscopy (DDS), and mechanical measurements. Characterization revealed that slowly cooled samples exhibited a significantly higher degree of crystallinity and larger crystallites (χ ≥ 55% and L_(110)_ ≈ 20 nm), compared to quenched samples (χ < 30%, L_(110)_ ≤ 3 nm). Mechanical testing showed a drastic contrast: quenched samples exhibited elongation at break > 500%, while slowly cooled samples broke below 15%, reflecting their brittle behavior. For the first time, DDS is applied to investigate molecular mobility differences between processing-dependent structural forms, specifically the mesomorphic (smectic) and α-monoclinic forms. In slowly cooled samples, α relaxation exhibited both enhanced intensity and an upward temperature shift, indicating stronger structural constraints due to a much higher crystalline phase content and significantly larger crystallite size, respectively. These findings provide novel insights into the structure–property–processing relationship, which is crucial for industrial applications.

## 1. Introduction

More than 50% of the thermoplastic polymers used in industry are semicrystalline (comprising both crystalline and amorphous phases), making crystallization essential to the material and component design process [1]. Crystallization and the presence of a crystalline phase play crucial roles in the microstructure, properties (optical, thermal, mechanical, etc.), and long-term stability [2,3]. PP is probably one of the best examples of how significant the presence of a crystalline phase is. The large difference between atactic PP (aPP), amorphous due to lack of any regularity preventing it from crystallization, and semicrystalline ones, characterized by greater structural regularity and the presence of a crystalline phase, illustrates this significance. Tacticity, or the overall stereochemical order of a polymer chain, is determined by the configuration regularity of successive stereocenters. In PP, the stereogenic center is the carbon atom that carries the methyl substituent. Strictly speaking, the presence of a crystalline phase in PP is directly related to the tacticity of the basic chain structure. In the context of PP, tacticity refers to the relative spatial arrangement of the methyl (−CH_3_) groups along the polymer chain and is commonly expressed as a percentage using the isotactic index. PP has three possible stereochemical configurations: atactic (aPP, without any regular order, in which case the methyl groups (−CH_3_) are randomly aligned), syndiotactic (sPP, with methyl groups on alternating sides of the chain), and isotactic iPP, with all methyl groups (−CH_3_) on the same side of the chain [4]. An increase in isotacticity leads to a semicrystalline PP with significant crystallinity and favorable thermal and mechanical properties [5,6,7,8]. The mechanical properties of PP, such as softening point, rigidity, Young’s modulus, strength, and toughness, are improved by increasing isotacticity [9,10,11]; the higher the isotacticity (i.e., the isotactic fraction), the greater the crystallinity, and thus also the mechanical properties [12]. Therefore, the most essential commercial PPs used in industry are isotactic ones [13,14,15], with isotacticity greater than 95% in many cases [9], and with a degree of crystallinity that in industrial products can be up to 60% [15,16].

Due to its considerable structural complexity, the great interest of the scientific community, and the wide range of different industrial applications, isotactic PP (iPP) has been studied extensively for its polymorphic characteristics and crystallization behavior since 1954, when Natta et al. synthesized it for the first time [17]. Polymorphic iPP can exist in several crystalline forms (modifications) that differ in the molecular arrangement of the polymer chains, and its crystallization is strongly dependent on the crystallization conditions and molecular characteristics. Thus, iPP is unique in adopting the same three-fold helical conformation with a 6.5 Å repeat distance in as many as three distinct crystalline polymorphs, monoclinic (α), hexagonal (β), triclinic (γ), and in the so-called mesomorphic (smectic) phase as well [18,19,20,21]. In all crystalline forms, within the lattice, the polymer chain adopts a 3_1_-helical conformation, either left- and/or right-handed, with the methyl groups positioned in an ‘up’ or ‘down’ orientation [22,23,24,25]. Crystallization from the melt under high undercooling or pressure, or in the presence of nucleating agents, can yield the β-hexagonal or γ-triclinic forms [18,22]. Accordingly, the β-form is promoted by nucleating agents, as well as by thermal gradients and shear stress, while the γ-form is rarely observed under standard industrial processing conditions and is facilitated by high-pressure crystallization or disruptions in the isotactic sequence length [23,26,27]. Rapid quenching of molten iPP does not yield a completely amorphous material and instead induces the formation of a mesomorphic (smectic) phase with intermediate crystalline order [22,28,29]. The mesomorphic phase is arranged roughly as in the α crystal, with defects along the chains and in the packing perpendicular to the chain direction [30]. This form is considered a reason for the transparency in rapidly cooled films (due to low order and small crystallites) and often occurs in industrial processing since the plastic is usually cooled quickly [31,32]. Under isothermal crystallization, in slow cooling and during melt spinning of filaments, iPP crystallizes mainly into the thermodynamically most stable monoclinic α-form [17,22,33]. This form often occurs in practice, and it is generally characterized by the highest crystallinity among other forms, which can exceed 50% in industrial products.

The crystal structure and crystallization kinetics of iPP are well established [34]. iPP crystallization is a critical process involving the organization of three-fold helical molecular chains. It has been shown that the rate of nuclei seeding is significantly influenced by tacticity, with a high-tacticity iPP sample exhibiting a faster nucleation rate [35]. At the nm scale (in most cases between 5 and 25 nm), the chains are arranged in lamellae, and the most common crystalline structures under melt processing conditions are lamellar-like crystallites [36]. At a larger (μm) scale, the initial nuclei, through lamellar propagation and multiplication, give rise to spherically symmetric superstructures known as spherulites [37]. The structure of spherulite was established by the pioneering work of Bassett and co-workers, where the notions of dominant and subsidiary lamellae were introduced and further investigated with a combination of light-microscope methods, the etching method, electron microscope (EM), and atomic force microscope (AFM) [36,38,39,40,41,42,43,44,45,46,47,48,49,50]. In general terms, a spherulite can be defined as a crystal aggregate with spherical symmetry formed by radial growth of crystals (stacked lamellae) originating from a common center [35,51]. According to Norton, such growth and the resulting spherically symmetrical end product, however, can arise in more than one way, most conveniently classified into two main categories: central multidirectional growth and sheaf-like unidirectional growth [52]. Depending on crystallization conditions, four different types of spherulites can be observed [42,45,52,53,54]; detailed descriptions of these structures, their characteristics, and conditions of formation are beyond the scope of this paper. The simplest morphological model of semicrystalline polymers is a two-phase model composed of crystalline and amorphous structures. However, between the pure amorphous (also known as the mobile amorphous fraction—MAF) and crystalline regions (lamellae and spherulites), an intermediate phase can be distinguished, in the literature known as the rigid amorphous fraction (RAF) or disordered crystalline phase [23,55,56,57,58,59]. The more ordered crystalline domains impose restrictions on the mobility of polymer chains within the interphase region. This region consists of the fold surface of the lamellae, chain loops, entanglements, knots, tie chains, and chain cilia [55,60]. While the molecular mobility of chains in the MAF, which are liquid-like amorphous chains, is released at the glass transition temperature, the molecular motion of chains in the RAF is constrained at T_g_ because of strong coupling with the crystalline structure [56].

Therefore, crystallization kinetics are of paramount importance in designing polymers for specific applications, as they influence the morphology developed during solidification and, consequently, the resulting properties. By adopting widespread melt processing techniques, such as extrusion, heat compression, film stretching/blowing, injection molding, and fiber spinning, distinctly different temperature and/or stress fields may be imposed on the melts of semicrystalline polymeric materials, leading to tremendous differences in the microscopic structures, crystallinity, and macroscopic properties of the final products [61,62]. A good example is commercial PP, which is mainly isotactic. Even with an unchanged chemical composition and molecular chain architecture, iPP can exhibit a wide range of final microstructures, different crystal architectures, and, consequently, large variations in macroscopic properties. Therefore, variation in processing conditions after melting not only determines how to achieve superior performance [63,64] and/or functionalization [65,66] of the final products, but also illustrates how the same polymer grade can be tailored for different applications by adjusting processing conditions. Processing conditions after melting, such as cooling rate and/or solidification temperature, play a significant role in obtaining isotropic polymeric structures. Thus, for iPP, it is well known that in the case of slow cooling rates, the crystalline α-form with spherulite morphology and a large content of crystalline phase (high crystallinity) is typical. In contrast, by quenching at room temperature, or, as it is better, at much lower temperatures, and/or by rapid cooling of molten PP at a rate of more than 10^2^ to 10^3^ K/s, the mesomorphic (smectic) phase can be obtained [55,67,68,69,70,71]. The mesomorphic phase has molecular ordering between that of the amorphous and of the true crystalline phase [55,72,73,74], with molecules arranged roughly as in the α crystal [55] but containing defects along the chains and in the packing perpendicular to the chain direction [30,75]. The effect of isotacticity on the mesomorphic phase-forming properties was investigated by Konishi et al. [74]. Under the same quenching conditions, iPP with higher isotacticity formed the mesomorphic phase, whereas iPP with lower isotacticity developed an α-crystalline form instead. Nevertheless, the structure of the mesomorphic phase remains unresolved. Although most researchers attribute the structure to the smectic phase, additional explanations are based on crystal defects: paracrystalline, conformational disorder (condis) crystal, and micro- or nanocrystal [74]. The mesophase structure can be transformed into the α-form with a nodular morphology by annealing at temperatures above 60 °C due to the ordering of the lateral direction of molecular chains [55,70,72,76,77,78,79]. The lamellar structure of the α-form crystals prepared from the mesophase only comprises the parent lamellar crystals [10]. The mechanism by which the meso-to-α phase transformation takes place has been investigated, and it has been reported that left- and right-handed helices occur during the annealing process [80,81]. The mesomorphic (smectic)-to-the-α-monoclinic phase transformation upon heating is possibly by thickening of existing α crystals and/or by structural rearrangements in the mesomorphic phase [82]; the mechanism of this structural rearrangement still has to be definitively established [22,80,83,84,85,86]. Since the formation of the RAF is evidenced for both mesomorphic and crystalline iPP, structural changes in the RAF seem to be very important for understanding the crystallization of the mesophase [58,72,79].

Processing conditions combined with specific structural characteristics can lead to products with large differences in final microstructure, crystallinity, and physical properties despite the starting material being the same. Thus, this work aims to shed some new light on the investigation of the influence of processing conditions on the crystalline architecture and final properties of commercial PPs. Two opposite cooling procedures after compression molding, rapid quenching and slow cooling, which represent border conditions in the case of industrial processing, were applied to obtain PP samples with great diversity in microstructure and properties. Microstructures and crystalline architectures were studied by optical microscopy (OM), scanning electron microscopy (SEM), Fourier transform infrared spectroscopy (FTIR), and wide-angle X-ray diffraction (WAXD). Differential scanning calorimetry (DSC) was used to study thermal properties. For the first time, dynamic dielectric spectroscopy (DDS) is employed to investigate the differences in molecular mobility between the iPP mesomorphic (smectic) and α-monoclinic phases. Special attention was given to tensile properties and stress–relaxation behavior to gain deeper insight into the relationship between structure and mechanical properties. Two additional PPs (one with nucleating and antistatic agents and the other with ionizing radiation stabilizers) are used in the mechanical measurements to confirm the relations between the mechanical behavior of the final structures and the applied processing conditions after compression molding. This work also presents a broad discussion of literature data in line with the new observed results presented in this paper.

## 2. Experimental Section

### 2.1. Materials

Two types of PP, PP-A (isotactic PP used in various industries obtained from SIGMA-ALDRICH, with a high index of isotacticity, density ρ = 0.90 g cm^−3^, M_w_ = 250,000, M_n_ = 67,000, more information at https://www.sigmaaldrich.com 5 December 2024) and PP-H (commercial PP HIPOLEN MA2CR intended for the production of goods for pharmaceuticals, cosmetics, and thin-walled containers with an index of isotacticity > 95% (≥98% according to ISO/DIS 1873-1), ρ = 0.91 g cm^−3^, M_w_ = 136,000, M_w_/M_n_ = 4.95, more information at https://hipol.com) were thoroughly examined. In an attempt to obtain a more complete picture of the influence of different preparation methods on the mechanical properties, two additional commercial PPs were tested, one with nucleating and antistatic agents and the other containing stabilizers against ionizing radiation. The first one is PP-T (TIPPLEN H 949 is a high-melt-flow PP homopolymer with outstanding processability for shorter cycle times, which contains nucleating and antistatic agents. Melt flow rate: 45 g/10 min, ρ = 0.90 g cm^−3^, more information at https://molgroupchemicals.com). The second one is PP-P (PP-PURELL HP 371P with a gamma ray-stabilizing additive for injection molding applications, primarily designed for disposable syringes for medical applications; melt flow rate: 18 g/10 min, ρ = 0.90 g cm^−3^, more information at https://www.matweb.com).

Isotropic sheets were prepared by compression molding for 8 min in a Carver laboratory press at 190 °C with a gradual pressure increment up to 3.28 MPa. The first set of samples (Q samples) was obtained by quenching in the ice–water mixture; the use of ice–water-quenched PP is recommended when increased transparency, impact strength, and flexibility are desired [32]. The second set was prepared by slow cooling from the melt to room temperature, keeping the samples between the press platens (SC samples). Next, depending on the measurement method, PP samples of various sizes were cut from isotropic foils of thickness 0.28 ± 0.02 mm and utilized in subsequent analyses.

### 2.2. Characterization Techniques

Microstructure characterization was performed using a Carl Zeiss “AxioImager A1” optical microscope and a JSM 5300 scanning electron microscope (JOEL Ltd., Tokyo, Japan). OM photomicrographs were captured and analyzed by a high-resolution microscopy camera (AxioCam, Carl Zeiss, Oberkochen, Germany) and image processing software (AxioCam, Carl Zeiss, Oberkochen, Germany). For SEM analyses, the surface of the samples was covered with a 10 nm thick layer of gold using a Polaron E5200 sputter coater (QuorumTechnologies Ltd., Laughton, UK). The obtained thickness was verified by profilometric measurements [87].

Fourier transform infrared spectroscopy of PP films was performed in attenuated total reflectance mode (ATR-FTIR). The spectra were recorded using a Nicolet 6700 spectrometer equipped with a diamond crystal attachment (Thermo Scientific, Waltham, MA, USA) at room temperature in the wavenumber range of 4000–400 cm^−1^ with a resolution of 4 cm^−1^. The resulting spectra represent the averaged values of three randomly selected, identically prepared samples. The crystal fraction (crystallinity) was determined using a procedure based on the Lanyi equation [88,89].

WAXD analyses were performed using a Philips PW 1710 diffractometer (Philips, Eindhoven, Netherlands). The data were collected in the 2θ = 5–90° range, with a step length of 0.03° and an exposure time of 4 s per step. The average out-of-plane crystallite size of the PPs (L) was estimated from the peak that corresponds to (110) reflection using the Scherrer equation: L = K λ/β cosθ, where λ is the wavelength of CuKα radiation (λ = 1.5418 Å), θ corresponds to the Bragg angle, β is the full width at half maximum (FWHM) in radians, and K is the coefficient, taken to be 0.89. The deconvolution of the X-ray diffractograms was performed using a Gaussian–Lorentzian–pseudo-Voigt asymmetric function to separate the amorphous (A_am_) and the crystalline (A_c_) content and calculate the crystallinity, crystallite size, and space between the structural layers. According to the profile fitting process, the share of the crystalline fraction was calculated as χ_c_ = A_c_/(A_c_ + A_am_) × 100% [90].

For the differential scanning calorimetry (DSC) measurements, a Perkin Elmer DSC-4 was used. Samples of 7–8 mg were analyzed by heating from 320 to 470 K at a rate of 10 K/min, and the peak melting temperature (T_m_), FWHM (full width at half maximum), melting enthalpies (ΔH_m_), and crystallinity (χ) were derived from the heating scans. The degree of crystallinity was calculated using ΔH_f_ = 209 J/g as the heat of fusion of a perfectly (100%) crystalline PP. In the case of PP samples with mesomorphic (smectic) phase, besides the enthalpy of melting (ΔH_m_), we also determined the enthalpy of cold crystallization (ΔH_C_), while the degree of crystallinity (χ) was calculated according to χ = (ΔH_m_-ΔH_C_)/ΔH_f_. DSC measurements were performed on five identically prepared samples of each kind, randomly selected. The presented results are average values.

Dielectric spectra of the samples in the form of disks, 1.3 cm in diameter, were measured on an Agilent 4284A Precision LCR Meter coupled with a 22C-kriodin(R) cryo-system, as a function of temperature (20–410 K) and in the frequency range 10^2^–10^6^ Hz. Dielectric measurements were taken at increments of approximately 2 K, with a heating rate of 0.5 K/min between equilibrated temperatures. At each equilibrated temperature, capacitance and tan δ measurements were taken at 24 frequencies, with an emphasis on 100 kHz and 1 MHz.

Tensile properties were measured using a Shimadzu AGS-10kN. Dumbbell-shaped specimens were cut from differently prepared, 0.28 mm thick, PP films using a cutter and had a gauge length of 30 mm and width of 4 mm. The unexposed and irradiated samples were elongated at room temperature (25 °C) with a constant elongation rate of 20 mm/min. The tensile stress was determined by dividing the tensile load by the initial cross-section, and the tensile strain was calculated from the ratio of the increment of the length between clamps to the initial gauge length. Young’s modulus was evaluated from the initial slope. The reported values of the investigated mechanical parameters are averages of the values obtained from 20 tensile bars of each material. Specifically, out of 24 initial values, the 2 highest and 2 lowest were excluded.

## 3. Results and Discussion

### 3.1. Microstructure Investigation

**Figure 1:**
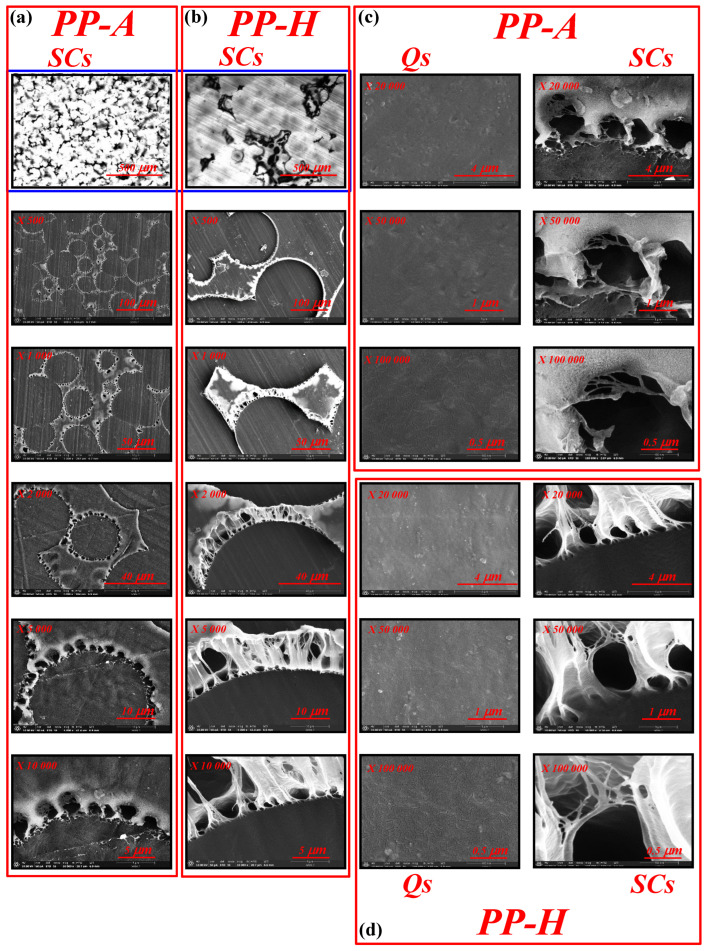
SEM images of SC surfaces for PP-A (**a**) and PP-H (**b**) at different magnifications (from ×500 up to 10,000). Images at the top, obtained by OM at magnification ×100; SEM images of Q (**left**) and SC (**right**) surfaces for PP-A (**c**) and PP-H (**d**) at large magnifications (from ×20,000 up to 100,000).

Microstructure comparison (with a gradual increase in magnification) of PP-A and PP-H samples obtained by slow cooling (SC) is presented in Figure 1a,b, respectively. OM images (presented at the top of Figure 1a,b) show spherically symmetrical superstructures, i.e., spherulites, among which the largest number are deformed. Significantly larger spherulites (d > 100 μm) are visible in the case of PP-H, indicating a higher potential of this material for the formation of large crystalline superstructures. Spherulite surface morphology, spatial boundaries, and defects are more clearly visible from SEM microstructures at intermediate magnifications (up to ×10,000) for both PPs. Higher SEM magnification (Figure 1c,d) also shows the presence of large cavities and fiber-like formations at the spherulite boundaries, which are more pronounced in PP-H. The presence of cavities at crystalline surfaces can be explained by the absorption of material from low-density (amorphous) regions during spherulite growth at very slow cooling conditions from the melt. On the other hand, fiber-like formations consisting of bundles of thinner fibers (which can be seen at high magnifications) represent inter-phase regions (RAF or disordered crystalline phase) with restricted mobility, which cannot be inserted into spherulites and contain a large concentration of tie chains, chain loops, etc. In contrast to the investigated PP samples obtained by slow cooling, in the case of samples obtained by rapid quenching (Qs), relatively smooth, non-porous surfaces with no signs of noticeable crystalline architecture and defects are visible even at high magnifications (×100,000). According to Androsch et al. [55], the absence of superstructure architecture (spherulites) due to quenching at rates faster than 10^2^ K/s to ambient temperature should be expected.

### 3.2. WAXD Study

**Figure 2:**
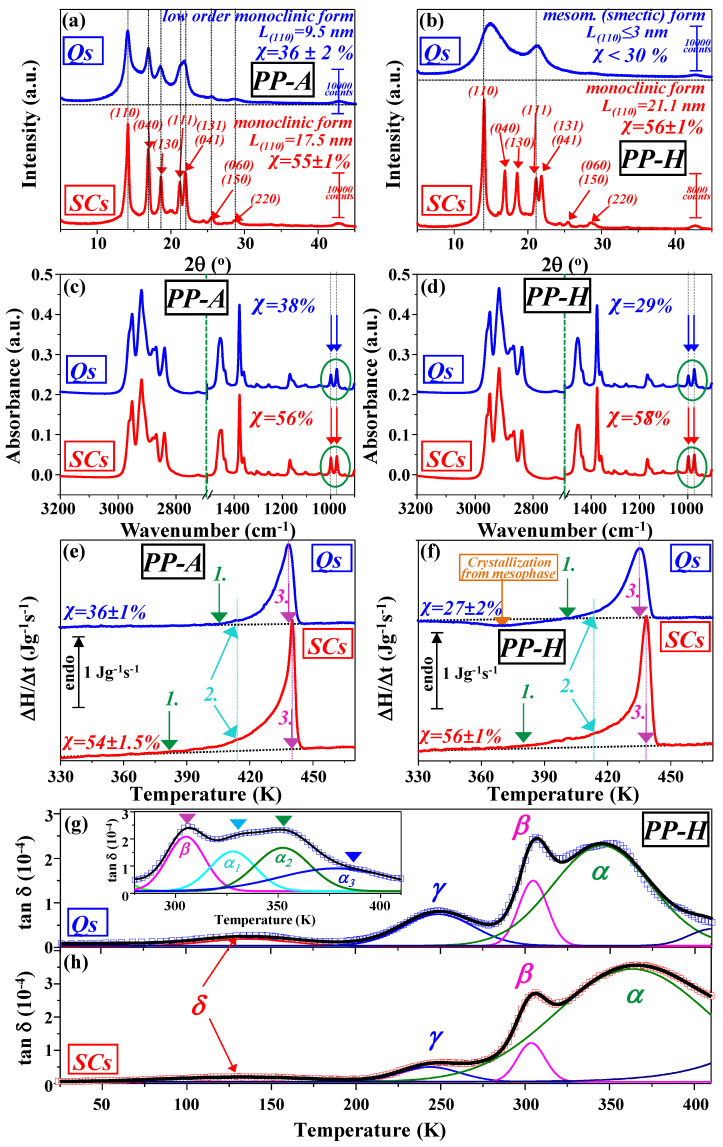
WAXD diffractograms of quenched (Q) and slowly cooled (SC) samples for PP-A (**a**) and PP-H (**b**) samples; ATR-FTIR spectra of Q and SC samples for PP-A (**c**) and PP-H (**d**) samples; DSC heating thermograms of Q and SC samples for PP-A (**e**) and PP-H (**f**) samples; experimental (□) and fitted (solid line) dielectric loss tangent spectra at 1 MHz for Q (**g**) and SC (**h**) PP-H samples. The insert in Figure 2g is the dielectric loss tangent spectra of the PP-H Q sample in the high-temperature α relaxation region.

WAXD patterns of PP-A and PP-H samples obtained after quenching in an ice–water mixture (Qs) and slow cooling from melt to room temperature (SCs) are presented in Figure 2a,b, respectively. A large number of narrow peaks (corresponding to (110), (040), (130), (111), (131), (041), (060), (150), (200), and (222) reflections) in the SC diffractograms clearly confirm the presence of a highly developed monoclinic (α) form for both PPs (PP-A and PP-H) [91,92,93]. Furthermore, the parameters related to the crystal phase obtained from the SC diffractograms are determined: PP-A (degree of crystallinity χ = 55 ± 1%, crystallite size L_(110)_ = 17.5 nm) and PP-H (degree of crystallinity χ = 56 ± 1%, crystallite size L_(110)_ = 21.1 nm). This data indicates that significantly larger spherulites in the case of PP-H, obtained previously by comparison of surface microstructures, do not lead to a significant variation in the shape of the diffractogram or the overall crystallinity (degree of crystallinity), since the difference is less than 2%. On the other hand, while the difference in crystallinity is relatively small, the crystallite size is about 20% larger in the case of PP-H.

While the slow cooling of different grades of commercial PP developed a monoclinic (α) form with high crystallinity, quenching in the ice–water mixture after melting shows significant differences in the obtained diffractograms. Thus, the same applied quenching procedure in the case of PP-H resulted in a clear mesomorphic (smectic) form characterized by two wide diffraction peaks, while in the case of PP-A it led to an intermediate form, i.e., a mixture of the monoclinic (α) form and mesomorphic (smectic) one. In the diffractograms of Q samples of PP-A, three peaks can be clearly noticed ((110), (040), and (130)), while a broad peak at 21.5 ° corresponds to (111), (131), and (041) reflections. On the other hand, microstructure investigations of Q samples of PP-A indicate the absence of spherulite morphology and a microstructure similar to that of mesomorphic (smectic) PP-H. Parameters calculated from the Q samples of the PP-A diffractogram give a degree of crystallinity χ = 36 ± 2% with crystallite size L_(110)_ = 9.5 nm. Rapid quenching of molten PP-H fails to produce a totally amorphous polymer, but rather leads to a mesomorphic (smectic) form. Although the X-ray scattering curve for the quench-cooled sample is very similar to that for atactic PP, the presence of a second scattering maximum at 21.3 ° suggests the existence of a greater degree of order [94]. Increased transparency and flexibility of quenched PP with mesomorphic (smectic) form is attributed to the low order and small crystallites [32]. While this phase is stable at room temperature for long periods, it is very sensitive to thermal treatment, and upon heating the mesomorphic transforms into the monoclinic form by thickening of existing crystals and/or by structural rearrangements in the mesomorphic (smectic) phase [22,28,77,95]. For Q PP-H samples, i.e., mesomorphic (smectic) form, it is not easy to determine the parameters related to the crystal phase from the diffractogram. However, calculations indicate that the degree of crystallinity is lower than 30%, while crystallite size L_(110)_ ≤ 3 nm. Given the abundance of small crystallites and low-crystallinity characteristic of the mesomorphic (smectic) form, coupled with the occurrence of only two broad peaks in WAXD spectra, FTIR and DSC are likely more appropriate methods for examining the crystalline phase.

### 3.3. ATR-FTIR Spectroscopy

FTIR spectroscopy has been widely used in the past to examine the conformational changes of iPP during crystallization [74,91,96,97,98,99]. Furthermore, it is proposed that tacticity in PP can be determined by FTIR, besides extraction in boiling heptane and 13C-NMR spectroscopy [4,100,101]. With the in situ IR microspectroscopic imaging technique, conformational ordering at the growth front of the spherulite of iPP is also studied during the isothermal crystallization process at different temperatures [39,102]. The mechanism of the meso-to-α transition of iPP and ethylene−propylene random copolymers was investigated by FTIR spectroscopy in detail by Di Sacco et al. [76].

Nevertheless, in this study, the focus is on the calculation of crystallinity from the presented ATR-FTIR spectra (Figure 2c,d) following the Lanyi equation, χ = 0.62 h_998_/h_973_ (where h_998_ and h_973_ are peak heights at 998 and 973 cm^−1^, respectively) [88,89]. Besides Lanyi et al., other comparable approaches for determining the degree of crystallinity via FTIR spectroscopy have already been published by Burfield et al. [103], Huy et al. [104], and Kilic et al. [105]. The peak at 998 cm^−1^ corresponds to the crystalline phase, and the height of this peak, according to literature data, grows linearly with the degree of crystallinity [103,106,107]. The peak at 973 cm^−1^ correlates linearly with the penetration depth of the IR-radiated volume and is independent of the degree of crystallinity [108,109,110]. Therefore, the ratio of the maximum peak heights of the peak at 998 cm^−1^ (h_998_) and 973 cm^−1^ (h_973_) is considered as a linearly proportional measure of the degree of crystallinity χ with slope 0.62 determined by Lanyi et al. [88,89]. Such calculated degrees of crystallinity χ from the presented ATR-FTIR spectra (Figure 2c,d) are for Q and SC PP-A samples 38% and 56%, while in the case of PP-H, they are 29% and 58%, respectively. These data are in very good agreement with those previously obtained by WAXD.

### 3.4. Calorimetric Study

Besides thermal properties, DSC measurements are also suitable for investigating the crystalline phase of thermoplastic polymers, such as PP. DSC heating scans (thermograms) of PP-A and PP-H samples obtained after quenching in an ice–water mixture (Qs) and slow cooling from melt to room temperature (SCs) are presented in Figure 2e,f, respectively. Regardless of the type of PP and applied treatment after melting, all samples exhibit distinct endothermic peaks attributable to the melting of the crystalline phase. Only the Q PP-H samples exhibit a broad exothermic peak, which can be ascribed to crystallization from the mesophase, i.e., low-temperature crystallization (into the α-monoclinic phase) of polymer portions that, on the macromolecular scale, are placed close to the initially present smectic crystal phase [111,112,113]. This transformation upon heating is by structural rearrangements in the mesomorphic phase and/or thickening of existing α crystals [82]. Since it occurs at much lower temperatures than melting, it is also known as cold crystallization. Thus, the endothermic peak observed in Q PP-H samples, occurring at significantly higher temperatures due to crystalline phase melting, arises both from the melting of the smectic crystalline phase existing at room temperature and crystalline domains produced upon heating. Accordingly, the crystallinity was determined following the methodology described in the Experimental Section. Taking this into account, the degree of crystallinity obtained from DSC measurements is determined for PP-A (χ = 54% for SC and χ = 36% for Q samples) and PP-H (χ = 56% for SC and χ = 27% for Q samples). Comparing this data with that obtained from WAXD and FTIR spectroscopy, a good agreement can be observed in the degree of crystallinity. However, the degree of crystallinity obtained from DSC measurements is calculated using the value ΔH_f_ = 209 J/g as the heat of fusion of a perfectly (100%) crystalline PP [114,115]. ΔH_f_ values reported in the literature can deviate more or less from this value, but in most cases, they are between 150 and 250 J/g [88]. Besides the degree of crystallinity, DSC measurements are used to determine the melting temperature (T_m_) of PP-A (438 K for Q and 440 K for SC samples) and PP-H (435 K for Q and 439 K for SC samples). Higher values of melting temperatures are revealed in slowly cooled (SC) samples, i.e., those with a fully developed α-monoclinic phase and spherulites, than in the case of quenched (Qs) (which are previously reported), and the obtained results are in good agreement with literature data, where T_m_ ranges from 433 to 439 K [9,20,116]. For comparison, perfectly crystalline iPP has a slightly higher value (T_m_ = 444 K), while sPP has lower values (423 K ≤ T_m_ ≤ 433 K) [117,118,119]. The shift to a higher melting temperature can be explained by the increase in crystallite size rather than crystallinity [120,121]. Namely, in the case of SC samples, the long period of the crystallites (i.e., stacking period, which is usually extremely regular) and lamellar thickening, which are favored by slow cooling after melting, lead to a large increase in crystallite dimension and the formation of very thick lamella with higher thermal stability and perfection [76,116,122,123,124,125,126,127]. In addition, Figure 2e,f indicate large differences in the shapes of the melting peaks. The first observation is related to the width of the melting peaks. The full width at half maximum (FWHM) for SC samples is between 5 and 6 K (5 K for PP-A and 6 K for PP-H), while in the case of Q samples, it is almost twice as large, between 9 and 11 K (9 K for PP-A and 11 K for PP-H). Combining DSC with WAXD data, it can be concluded that the smaller (larger) the crystallites, the broader (narrower) the melting peak, revealing a large distribution of crystal defect concentration and size in the crystalline core of Q samples [20]. The second observation regards temperatures at which endotherms start to separate from the baseline (green arrows (1) in Figure 2e,f). These onset temperatures are shifted 20–25 K towards lower values in the case of SC samples compared to Q samples, but even in this case, they are located above 375 K. As we know, this phenomenon has not been discussed in the literature so far, and its interpretation is not simple. In the case of SC samples, an increase in crystallite size and degree of crystallinity consequently leads to a larger presence of a low-temperature component in the melting endotherm, probably due to the large diversity in the RAF segment (in concentration and size) containing strained molecules. The enthalpy determined between points 1 and 2 on the thermograms also indicates a significantly larger presence of these rigid amorphous (RAF) segments in the case of SC samples compared to Q ones. Since this fraction starts to play a role at temperatures higher than 100 °C, it can be, at least partially, connected to increased chain mobility in the interphase, i.e., softening of the RAF, and the reduction in stress at the points of entry of tie chains into the lamellae, as a result causing a partial release of accumulated energy. Based on these observations, it can be concluded that very slow cooling after melting in commercial PP leads to the formation of the α-monoclinic phase, with large spherulites, a higher crystalline content, and well-ordered large crystallites with a more uniform size distribution. Additionally, the melting endotherm reveals a notable presence of a low-temperature component, likely due to the diverse RAF segments in both concentration and size, constrained by molecules from the well-developed crystallites organized within crystalline superstructures, such as spherulites. Thus, fiber-like formations with restricted mobility, previously observed at the spherulite boundaries of SC samples, that cannot be inserted into the spherulites and are mostly composed of tie chains, chain loops, etc., can be connected with the RAF containing a bundle of strained molecules. At elevated temperatures, increased molecular mobility in the RAF phase will result in a reduction in stress at the points of entry of tie chains into the lamellae and consequently at least partial reduction in stress at the points of entry of fiber-like formations into spherulites. However, this will take place at much lower temperatures than those required for the melting of the crystalline phase.

### 3.5. DDS Study

Dynamic dielectric spectroscopy (DDS), also known as dielectric relaxation spectroscopy (DRS), is a powerful tool for studying the structure and molecular mobility of dipolar polymers [5,28,128,129,130,131,132,133,134,135,136,137,138,139,140,141,142,143,144,145,146,147,148]. PP, as well as PE (another thermoplastic member of the polyolefin (PO) family), is basically nonpolar [149,150,151]. However, the measurable dielectric (loss) signal arises since the material is invariably somewhat oxidized, containing polar groups such as carbonyl, peroxy, or hydroperoxy moieties, which can be considered tracer groups reflecting the motion of the polymer chains [147,148,152,153]. The presence of impurities (residual catalysts, antioxidants, etc.) also increases dielectric response [152]. Due to its low polarity, good mechanical properties, and heat resistance, PP has been widely used as electrical insulation, e.g., for cables and as a dielectric in power capacitors [154,155,156,157,158]. This is another reason for growing interest in the investigation of dielectric phenomena of PP-based materials [159,160]. DRS, together with thermally stimulated discharge current (TSDC) measurements, can also be used to assess oxidative degradation and deterioration in PO cable insulation [28,161,162,163]. Considering certain specific factors, the investigation of polymer chain motion and thermodynamic transitions using DRS generally aligns with the findings of dynamic mechanical analysis (DMA), which is most commonly applied to PP [141,164,165].

In relaxation studies, PP exhibits four mechanical/dielectric relaxations, designated as α, β, γ, and δ—in order of decreasing temperature—in addition to its melting point [5,28,129,130,131,132,133,134,135,136,137,138,139,140,141,142,143,144,145,146,166]. The phenomena underlying these relaxations have been previously investigated, primarily using mechanical measurements, although some dielectric studies have also been reported [5,128,130,131,132,133,134,135,136,137,138,139,140,141,142,143]. While certain detailed molecular interpretations remain a subject of debate, the fundamental aspects of the basic relaxation processes are widely accepted. The α and β relaxations are clearly related to the crystalline and amorphous phases, respectively. According to Jourdan et al. [135], α relaxation arises from the relaxation of defects in the crystalline phase, although the contribution of the rigid amorphous fraction (RAF) has also been shown. This relaxation exhibits a complex character, comprising two or more distinct processes in the α relaxation zone [28,139,145]. β relaxation is associated with the glass transition within the amorphous regions of iPP. Various researchers attribute γ relaxation to localized, presumably crankshaft-type, movements of chain ends or branches within the amorphous phase [137,139,141], though it was initially also suggested to originate from the crystalline phase. In dielectric relaxation measurements, iPP can display a fourth relaxation, termed the δ process, which is primarily observed under 100 K. Attributed to the hindered rotation of CH_3_ groups, this relaxation is generally weak or may be absent [129,143].

In the case of PP, to the best of our knowledge, DRS has not been used to investigate the structure and relaxation of slowly cooled (SC) samples. However, we have previously used DRS to investigate differently modified quenched (Q) samples [144,149,167]. Namely, due to the wide application of PP in medical devices and the necessity for ionizing radiation sterilization of such products, DRS was previously used successfully not only for investigation of structural relaxation in the mesomorphic (smectic) phase but also to obtain valuable information about the level of overall oxidation and distribution of oxidation species within regions with different ordering [16,28]. Herein, to investigate the influences of different processing conditions, dielectric loss spectra for Q and SC samples are presented in Figure 2g,h, respectively. The presence of three relaxations can be clearly confirmed—high-temperature α relaxation (located around 350 K for Q and at 370 K for SC samples), β relaxation (around 300 K), and low-temperature γ relaxation (around 250 K). The presence of the fourth δ relaxation, which occurs at temperatures below 150 K, can also be confirmed. Still, this relaxation is barely noticeable, especially in the case of SC spectra, and will not be discussed further.

Prior to the discussion, it is important to note that SC samples are subject to extended exposure to high temperatures during slow cooling from the melt and consequently can have a slightly larger number of oxygen-containing groups in the structure due to thermal degradation [168]. On the other hand, dielectric loss spectra are sensitive to even a minimal increase in polar groups in the molecular structure of apolar polymers such as PP [87]. By comparing the dielectric loss spectra of Qs and SCs in the case of γ relaxation, only a difference in intensity is observed, and it is much larger in the case of Q compared to SC samples. Otherwise, shape, position, and determined activation energy (35–40 kJ/mol) are only slightly affected. Since this relaxation originates from the amorphous phase, it is expected that an increase in the degree of crystallinity, as is the case with SC samples, will lead to a decrease in the intensity of this relaxation. The dynamic mechanical investigation of iPP thermo-oxidative degradation indicated that the initiation of thermal oxidation is concomitant with a partial vanishing of γ relaxation [132,137,145]. Additionally, dielectric γ relaxation was found to vanish entirely after gamma irradiation in air [144,149] and similarly under ultraviolet radiation [131,138], confirming its intense sensitivity to oxidative degradation.

Further comparison of the dielectric loss spectra of Q and SC samples indicates that the position of β relaxation is almost unaffected, while α relaxation shows a significant shift to higher temperatures in the case of SC samples (370 K) compared to the Q samples (350 K). In addition, a significantly broader α peak can be observed in the SC samples compared to the Q ones, while the peak shapes of β relaxation are the same. Furthermore, a higher intensity of α relaxation is observed for SC samples, while the ratio between the intensities of α and β relaxation (I_α_/I_β_) increases from 1.5 (for Qs) to almost 3 (for SCs), favoring α relaxation increase in the case of SC samples. All of these features can be associated with large differences in the degree of crystallinity and crystallite size between Q (i.e., mesomorphic (smectic) form previously characterized by low content of crystalline phase with small crystallites) and SC samples (i.e., developed monoclinic form previously characterized by high content of crystalline phase with large crystallite size and developed spherulites). Hence, the enhanced intensity and the upward temperature shift in SC samples can be attributed to a much larger crystalline phase content and significantly larger crystallite size [76]. Such behavior of α relaxation can be well related to the behavior of the crystalline phase during melting obtained by DSC measurements. Even a significantly broader α peak in the case of SC samples can be attributed to the increased presence of low-temperature components in the melting endotherm, probably due to the wide dissipated distribution of the RAF segment (in concentration and size) containing strained molecules.

Furthermore, in the case of dielectric loss spectra of the Q sample presented in Figure 2g, experimental data were fitted with substantial success utilizing only one α relaxation peak, while the insert in Figure 2g represents the same experimental data in the α region much better fitted with three split peaks, confirming the complex nature of α relaxation in the mesomorphic (smectic) phase, consisting of two or even more processes. Such α relaxation behavior in the case of mesomorphic (smectic) form is also confirmed by DMA; according to Seguela et al., α_1_ relaxation, which occurs around 330 K, indicates higher energy absorption due to more intense molecular mobility and can be connected with cold crystallization, i.e., crystallization from the mesophase [20]. Activation energies for β and α relaxation are calculated by Vogel–Fulcher–Tammann–Hesse (VFTH) and Arrhenius equations, respectively, following the approach outlined in more comprehensive detail in previous studies [149]. Obtained differences in activation energies between Q and SC samples are relatively small for both relaxations and, in the case of β relaxation, range from 480 to 560 kJ/mol (with dynamic fragility m from 98 to 105), while for α relaxation, they are in the interval from 90 to 110 kJ/mol. Results obtained from dielectric loss spectra also indicate that the restriction in movements and reorganization of chains associated with the crystalline phase are reduced at elevated temperatures. The occurrence of α relaxation is undoubtedly connected with the crystalline phase, and it is due to relaxation of defects in the crystalline phase. The rigid amorphous fraction (RAF) also contributes to this process. However, its relaxation occurs at significantly lower temperatures than those required for the melting of the crystalline phase.

### 3.6. Mechanical Study

Among its physical properties, the mechanical properties are arguably the most important, making PP suitable for a wide range of applications, from medical and pharmaceutical to packaging and automotive. The mechanical properties of PP, as a plastic or a fiber, are widely investigated due to its sizeable structural complexity, the existence of different stereochemical configurations, polymorphic nature, different processing conditions, exposure to different external conditions, stretching and fiber applications, radiation sterilization of medical devices, recycling, blending with other polymers, application in composites, etc. Nevertheless, several new studies in which the mechanical properties of PP are in focus indicate that this area is still of great interest not only to industry but also to the academic community [23,159]. In the introductory section, we discussed the mechanical properties of PP and the influence of isotacticity. In general, the mechanical properties of PP, such as softening point, rigidity, Young’s modulus, strength, and toughness, are improved by increasing the degree of isotacticity as well as with the increase in crystallinity by changing processing conditions [9,10,11]. Herein, the focus is on the influence of processing conditions on commercial PP with high isotacticity. Samples obtained by two opposite cooling procedures after compression molding, rapid quenching and slow cooling, which represent boundary conditions in the case of industrial processing, are subject to investigation of tensile properties and stress–relaxation mechanical behavior in an attempt to gain better insight into the structure-to-mechanical property relations.

**Figure 3:**
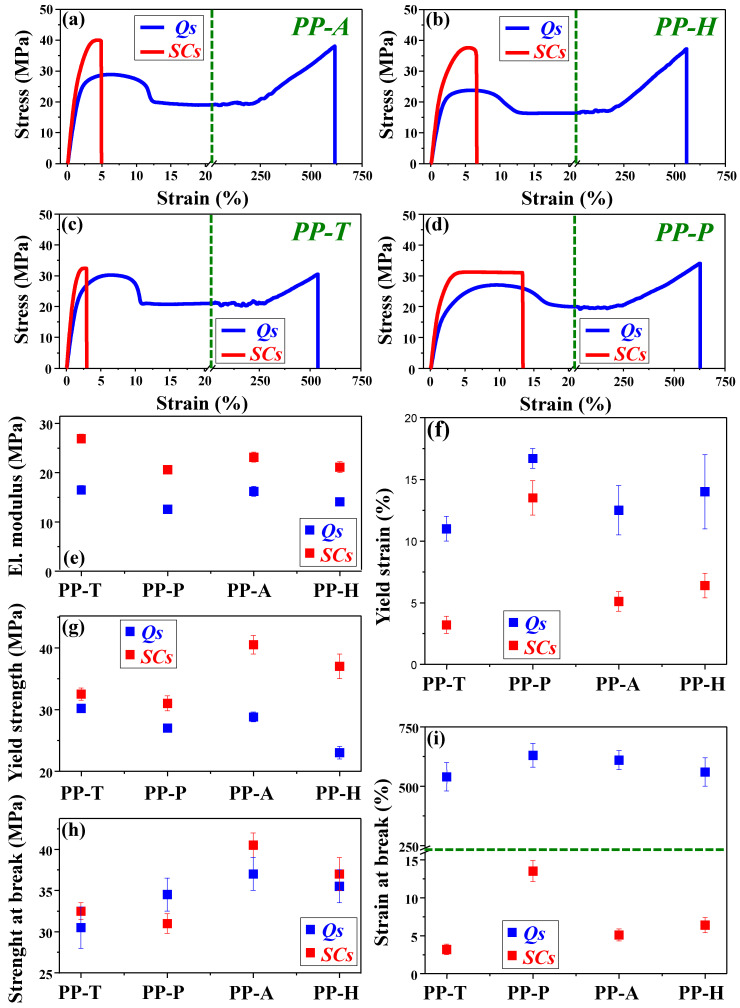
A typical tensile stress–strain diagram of Q and SC samples for four different PPs: (**a**) PP-A, (**b**) PP-H, (**c**) PP-T, and (**d**) PP-P; variation for Q and SC samples in elastic modulus (**e**), yield strain (**f**), yield strength (**g**), strength at break (**h**), and strain at break, i.e., total elongation (**i**), depending on PP type.

Typical tensile stress–strain diagrams of Q and SC samples for four different grades of commercial PPs are presented in Figure 3: PP-A (a), PP-H (b), PP-T (c), and PP-P (d). Analyzing the stress–strain curves for SC samples was challenging because strips (dumbbell-shaped specimens) were very fragile. An initial examination of Figure 3a–d shows that SC sample stress–strain curves resemble those of brittle materials, unlike the curves of Q samples, which are typical for ductile materials. Namely, the curve of SC samples does not show the yield point and plastic deformation stage, thus indicating that slowly cooled commercial PPs exhibited brittle behavior in general. While Q samples undergo elastic as well as plastic deformation and elongate more than 500% before breakage, SC samples show only elastic deformation, and in the vicinity of the yield point (i.e., initiation point of plastic deformation), they break with elongations of less than 15% (Figure 3i). Total absence of plasticity in SC samples, evident from the obtained stress–strain curves at room temperature, is more or less expected since most literature data for highly crystalline (slowly cooled and annealed) iPP, with some exceptions [9,169], confirm brittle behavior with missing or poor plasticity [5,170,171]. As the temperature at which stress–strain measurements are performed gets larger, plasticity appears [172]. This can be explained by the fact that in highly crystalline iPP at room temperature and temperatures that are close enough to the glass transition temperature (T_g_), structural restrictions caused by high crystallinity, large crystallites, and the presence of spherulites act as if it is still in the glassy state. In the case of Q samples, despite the differences in the intermediate form, crystallinity, and crystallite size (which are much lower than in the case of SC samples) between PP-A and PP-H samples, very similar stress–strain behavior is observed; at room temperature, Q samples show intensive plastic deformation. After propagation of plastic instability (necking) over the whole sample length, strain-hardening occurs as a result of chain unfolding and orientation involving the so-called fibrillar transformation. Two different mechanisms of plastic deformation of crystalline polymers have been proposed in the literature and thoroughly discussed by Makarewicz et al. [173]. The first one is based on the phenomenon of emission of dislocations from the edges of the lamellar crystals and their movement within crystals via crystallographic slips, which was proposed by Peterson [174,175]. The second mechanism of yielding of semicrystalline polymers assumes noncrystallographic changes of the initial crystalline skeleton, leading to the formation of completely new crystalline structures dependent mainly on the deformation temperature. Peterlin and co-workers proposed the micronecking model, which changed the crystal lamellae from a folded morphology into a partially unfolded fibrillar one [176,177]. According to this model, plastic deformation consists of three stages in the cold drawing of crystalline polymers: the plastic deformation of the original spherulitic structure, the discontinuous transformation of the spherulitic into fiber structure by micronecking, and the plastic deformation of the fiber structure. Because of their commercial importance, much work has been directed toward understanding the cavitation and plastic deformation of semicrystalline polymers, as well as fiber structures [112,178,179,180,181,182,183,184,185,186,187,188,189,190,191,192,193]. Stress–strain measurements were also used to investigate the transformation of the mesomorphic (smectic) phase into the monoclinic (α) phase by annealing and the difference between these phases [5,9,20,169,171]. According to some of them [9,20,169,171], despite the presence of the monoclinic (α) phase with spherulites, annealed iPP specimens after quenching possess plastic deformation. This is probably due to an overall lower degree of crystallinity, thinner crystallite lamellae, and much smaller spherulites than in the case of slowly cooled samples from a melt.

Mechanical parameters are presented in Figure 3e–i to better understand how processing conditions influence the structure-to-mechanical property relations for commercial PPs. Thus, the elastic (Young) modulus, yield strain, yield strength, strength at break, and strain at break (elongation), determined from the stress–strain curves, are presented as a function PP type for Q and SC samples. As can be seen from Figure 3e,g, Young’s modulus and yield strength are higher in SC samples due to larger crystallinity and a more developed crystal architecture than in the case of Q samples. In the case of Q samples, the strength at break increases due to the formation of a well-developed fibrillar structure, with polymer chains preferentially oriented along the stretching direction [112], and can exceed the yield strength of the corresponding SC sample; this is evident for PP-P, i.e., PP with ionizing radiation stabilizers. In general, the presence of a nucleating agent can increase the crystallization rate and decrease the spherulite size of PP [194]. However, commercial polypropylenes with specific additives (PP-T and PP-P) show similar behavior and fit well with others within this study.

## 4. Conclusions

Due to their complex and hierarchical structure and the variety of industrial processing techniques, semicrystalline polymers such as PP require more detailed systematic studies, especially those based on new approaches or experimental techniques. In this work, two contrasting cooling procedures—rapid quenching and slow cooling—were applied after compression molding, representing boundary conditions relevant to industrial processing. The differences in microstructure, crystallinity, and physical (especially mechanical) properties were significant, despite utilizing the same commercial PP with a high isotactic fraction as the starting material. There are numerous investigations that only partially cover different segments of this topic. The lack of a systematic study was the primary motivation for investigating this topic in greater detail. The obtained results are generally consistent with the comprehensive literature data, although some segments remain contradictory.

While the slow cooling after melting of different grade commercial PPs results in a monoclinic (α) form with a well-developed spherulite texture, high degree of crystallinity, and large crystallites, quenching in an ice–water mixture after melting fails to produce a completely amorphous structure. Depending on PP grade, quenching yields a clear mesomorphic (smectic) form in one case and a more ordered but still intermediate form consisting of monoclinic (α) and mesomorphic (smectic) forms in another case. Surface microstructures, studied by various microscopies (OM and SEM), in the case of SC samples show large spherulites with large cavities and fiber-like formations at the spherulite boundaries, in contrast to the relatively smooth non-porous surface with no signs of significant crystalline architecture in the case of Q samples. WAXD was used to determine the parameters of the crystalline architecture, revealing in the case of SCs a significantly higher degree of crystallinity (χ ≥ 55%) and crystallite size (L_(110)_ = 17.5 nm and L(110) = 21.1 nm, depending on the PP type) in comparison to Q samples (for mesomorphic (smectic) form χ < 30% with L_(110)_ ≤ 3 nm, while for more ordered but still intermediate form χ = 36% with L_(110)_ = 9.5 nm). Thus, the results for the degree of crystallinity are in good agreement with those obtained from FTIR spectroscopy and DSC thermograms. The higher melting temperature in the case of SC samples is explained by lamellar thickening, which is favored by slow cooling after melting, leading to a large increase in crystallite dimension and the formation of very thick lamella with higher thermal stability and perfection. The presence of a low-temperature component in the melting endotherm of SC samples is attributed to the large diversity of RAF segments (both in concentration and size) containing strained molecules. To the best of our knowledge, DDS is used for the first time to study the difference in molecular mobility between the mesomorphic (smectic) and α-monoclinic phase of iPP. The two most important relaxations are investigated: β relaxation (glass transition), associated with the amorphous phase, and α relaxation, related to the crystalline phase. The latter arises from the relaxation of defects within the crystalline phase, with an additional contribution from the rigid amorphous fraction (RAF). Differences in the dielectric loss spectra are successfully correlated with the significant variation in the degree of crystallinity and crystallite size between Q and SC samples. Thus, the increase in α relaxation intensity and the shift in location to higher temperatures for SC samples are explained by a much larger crystalline phase content and significantly larger crystallite size, respectively. DDS data in the α relaxation region also confirmed that the restrictions on chain movements and reorganization associated with the crystalline phase are reduced at elevated temperatures.

Mechanical properties, probably the most important for widespread industrial applications of PP, are strongly influenced by different preparation methods, such as rapid quenching and slow cooling after melting. At room temperature, Q samples show stress–strain curves typical of ductile materials and undergo both elastic and plastic deformation, with elongation larger than 500% before failure. On the other hand, slowly cooled (SC) samples show typical brittle behavior, characterized by elastic deformation followed by failure in the yield region, with elongations of less than 15%. This can be explained by the fact that in SC samples at room and other test temperatures close enough to the glass transition temperature (T_g_), structural restrictions caused by high crystallinity, large crystallites, and the presence of spherulites act as if they are still in the glassy state. Young’s modulus and yield strength are significantly higher in SC samples due to the larger crystallinity and more developed crystal architecture than in the case of Q samples. In the case of Q samples, the strength at break increases due to the formation of a well-developed fibrillar structure, with polymer chains preferentially oriented along the stretching direction, and can exceed the yield strength of the corresponding SC samples. The stress–strain behavior of PPs with specific additives (one with nucleating and antistatic agents and the other with ionizing radiation stabilizers) is in good agreement with other investigated PPs.

The comprehensive, multi-technique approach, which combines microstructural analysis, crystallographic studies, and spectroscopy with calorimetric, DDS, and mechanical studies, provides a holistic view of polypropylene’s behavior under various processing conditions. The insights gained from comparing polypropylene samples subjected to rapid quenching and slow cooling provide valuable guidance for tailoring material properties through controlled processing conditions. This knowledge is directly applicable in various industrial sectors, including packaging, automotive, and biomedical fields, where the mechanical performance, thermal stability, and structural characteristics of polypropylene are critical. By understanding how cooling rates affect crystallinity, morphology, and mechanical behavior, manufacturers can optimize processing protocols to achieve the desired product attributes, thereby enhancing durability, performance, and functionality in final applications. Thus, this study bridges fundamental microstructural analysis with practical manufacturing considerations, thereby contributing to the advancement of PP processing technology.

## Data Availability

The original contributions presented in this study are included in the article. Further inquiries can be directed to the corresponding authors.
